# A Comparative Evaluation of ELISA, PCR, and Serum Agglutination Tests For Diagnosis of Brucella Using Human Serum

**Published:** 2017-10-01

**Authors:** Khashayar Mohseni, Reza Mirnejad, Vahab Piranfar, Shiva Mirkalantari

**Affiliations:** 1 *Student Research Committee, Semnan University of Medical Sciences, Semnan, Iran*; 2 *Molecular Biology Research Center, Baqiyatallah University of Medical Sciences, Tehran, Iran*; 3 *Microbiology Dept, Islamic Azad University of Tonekabon, Tonekabon, Iran*; 4 *Microbiology Dept, Semnan University of Medical Sciences, Semnan, Iran*; 5 *Microbiology Dept, Iran University of Medical Sciences, Tehran, Iran*; 6 *Institute of Immunology and Infectious Disease, Iran University of Medical Sciences, Tehran, Iran*

**Keywords:** *Brucella*, PCR, ELISA, Agglutination test, Human, Serum

## Abstract

**Background & Objective:**

Since the symptoms of Brucellosis are often atypical and nonspecific, using clinical signs alone to diagnose brucellosis is not advised; therefore, the diagnosis relies predominantly on laboratory testing. Currently, molecular, serological, and microbiological methods are used for diagnosis of this disease. In this study we examined ELISA, PCR and serum agglutination (SAT) methods on human patient serum samples.

**Methods:**

A total of 100 serum samples were collected from suspected patients. Fifty serum samples gave a positive result with the Wright test. The ELISA method was first employed on all samples for the detection of IgG and IgM antibodies against *Brucella*. Subsequently, the rapid PCR methodology was used to identify presence of *Brucella* genome in 500 µL of each serum sample. The B4/B5 primer pair was used for PCR‌ amplification.

**Results:**

Out of the 100 serum samples obtained from patients with suspected brucellosis, 50 samples tested positive by SAT and displayed high titers of 1/160. Of these 50 positive samples, 49 samples were positive as per the ELISA test whereas one sample tested negative. The PCR test was conducted on all 100 serum samples and results showed that the 45 serum samples that gave a positive agglutination test were also positive by PCR.

**Conclusions:**

Various laboratory methods have been used or introduced for the detection of *Brucella*. Molecular methods such as PCR, a rapid and sensitive method for detection of bacteria, have also been reported. Based on the results of this study, we propose that the simultaneous use of serology and molecular techniques has the potential to overcome limitations of detection thereby enabling the selection of appropriate treatment for the patient.

## Introduction

Brucellosis is one of the most common diseases that afflicts both humans and animals. It is prevalent in many regions of the world including Latin America, Middle East, the Mediterranean basin, Africa and Asia (1-3). According to WHO, more than half a million new cases of infection are reported in the world annually. *Brucella* can be transmitted to humans in several ways including the consumption of unpasteurized dairy products, inhalation of the microorganism as well as transmission through the skin. Since the clinical symptoms of human brucellosis are different and nonspecific, diagnosis of brucellosis is based on a positive assessment in laboratory-based testing. There are various assays available for diagnosing Brucella in humans and currently, molecular, serological, and microbiological tests are popularly used to the purpose. Blood culture is a gold standard method for *Brucella* detection but this method is time-consuming, increas the risk of disease transmission to humans, and suffers from an acute phase sensitivity of only 15 to 70% (4-8). In addition, it requires a high level of skill and safety parameters. Serological detection methods such as the Rose Bengal and the test tube agglutination test, commonly conducted in diagnostic laboratories, are also very common. In both Rose Bengal and tube agglutination, it is well known that lipopolysaccharides display cross-reactivity with the Gram-negative bacteria. In one study, conducted by Osoba, ELISA was reported as the rapid and reliable diagnostic test for brucellosis (1). The ELISA test has the several advantages, it can also detect the incomplete antibodies commonly observe in chronic brucellosis patients (9). 

Molecular methods are also used for the detection of bacteria in blood and serum samples. PCR has the ability to detect a very low level of bacteria in the sample and hence widely used as a tool for the diagnosis of infectious diseases. Despite the specificity of the method, its sensitivity has been reported to vary from 50 to 100% (7, 10-13). On the other hand, some studies have shown that serum samples are preferable to blood sample, as it can increase the sensitivity of PCR (14). In recent years, several studies have been done on the comparison and evaluation of various laboratory methods used for *Brucella* detection but the results are very variable. In this study, we have used the ELISA and PCR-based methods for the diagnosis of *Brucella* in serum samples isolated from patients with suspected infection.

## Materials and Methods

**Serum sample collection**

This cross-sectional study was conducted with proper approval from the Ethics Committee of the Semnan University of Medical Science (no. 868). A total of 100 serum samples were collected from patients with suspected brucellosis; these patients presented with symptoms such as headache, fever, chills, fatigue, joint pain, lower back pain, and back pain. Samples were collected from the laboratories of several sites including hospitals based in Tehran, Shahrekord, and Semnan. The specimens were collected between June and Aug 2015.

**Serum Agglutination Test**

All samples were transferred to the lab and tested by reacting against the *Brucella* antigen. Briefly, serum samples were diluted with sodium chloride solution prior to being mixed with an equal volume of *Brucella abortus* antigen. After incubation at 37 °C for 24 h, the samples were examined for the presence of agglutinin particles (15). Serum samples with titers greater than 1/80 were selected for the purpose of this study. Samples were then aliquoted into micro tubes and stored at –20 °C until required for testing with the PCR and ELISA methods. 

**ELISA detection test**

The ELISA protocol was conducted in accordance with the manufacturer's instruction (IBL International GmbH, Germany). All samples were analyzed for the presence of IgG and IgM antibodies against *Brucella*. On basis of protocol, 100 µL solution was used to dilute patient serum sample in the ratio 1:101. 50 ul of the diluted serum sample was then added to the ELISA plate and incubated for 60 min at room temperature. After washing, 100 µL of the enzyme conjugate was added to each well and incubated at room temperature for 30 min. TMB substrate solution (100 µL) was added and the plates were incubated for a further 20 min at room temperature. Stop solution was added to the reaction and the color action was read at 450 nm using an ELISA reader.

**PCR detection test**

A commercially available DNA extraction kit (QIAamp Blood Midi, QIAGEN GmbH, Hilden, Germany) was used to extract the *Brucella* genome from 500 µL of patient serum samples. Primer pair B4/B5 (described previously by Bailey *et al.*) was selected for DNA amplification by PCR (9)*. *The primer sequences were used as follows: Forward- 5’-TGGCTCGGTTGCCAATATCAA–3’ and Reverse- 5’-CGCGCTTGCCTTTCAGGTCTG–3’. The mentioned PCR assay amplified a 223-bp sequence of the *B. abortus* genome (*bcsp31*), which encodes an immunogenic outer membrane protein of 31 kDa. This region of the genome was known to be conserved in all *Brucella* species (9).

 The PCR reaction was used in a total volume of 50 µL. The reaction mixture was composed of the following: (a) forward and reverse primers at a concentration of 0.5 µM each, (b) 0.5 U of Taq polymerase, (c) 0.2 mM each dNTP, (d) 1.5 mM MgCl_2_, (e) 10 μL of template DNA (150 ng/mL) and, (f) 1× PCR reaction buffer. The PCR reaction was performed using a thermocycler (Eppendorf, Germany). PCR cycling conditions were performed as follow: initial denaturation of 95°C for 5 min followed by 40 cycles of 95°C for 1 min, 60°C for 30s, and 72°C for 30 min. This was followed by a final extension step of 72°C for 5 min. The presence or absence of the PCR product (10 µL from each reaction mixture) was determined by agarose gel electrophoresis (2% w/v) at 80 V for 45 min; gel was stained with ethidium bromide (0.5 µg/mL) and examined under a UV transilluminator. Pure water and DNA extracted from* Brucella melitensis* 16M were considered as a positive and negative controls respectively.

**Statistical analysis**

Degree of significance was calculated using chi-Square test. *P*-value less than 0.05% was considered significant. Statistical analyses were conducted using the SPSS version 16, (Inc, Chicago, IL, USA).

## Results

**Sample collection**

Of the 100 samples that were collected from patients with suspected brucellosis, 50 samples were positive in tube agglutination test conducted in the hospital laboratory. These 50 positive samples included 30 male and 20 female patients. The age of patients ranged between 15 days to 78 years with the average age of 40 years.

**Serum Agglutination test**

Fifty (50%) serum samples had positive results in the agglutination test whereas the remaining 50 samples were negative. All the 50 serum samples that tested positive in the agglutination test had titers greater than 1/160. The remaining 50 serum samples (those that gave a negative result in laboratory agglutination test) were considered as negative for Brucellosis. 

**ELISA detection results**

An enzyme-linked immunosorbent assay (ELISA) aimed at detecting anti *Brucella* IgG and IgM antibodies was also used to test the 100 patient samples that had previously been examined by the tube agglutination test. Of the 50 samples that had positive tube agglutination results, 49 were also (98%) positive in the ELISA test. Thirty (60%) serum samples were found to contain IgG antibodies while 40 (80%) had IgM antibodies against *Brucella*; 21 (42%) samples were found to have both IgG and IgM antibodies. The 50 serum samples that were negative with the agglutination tube test were also negative in ELISA test.

**PCR detection results**

**Figure 1.**
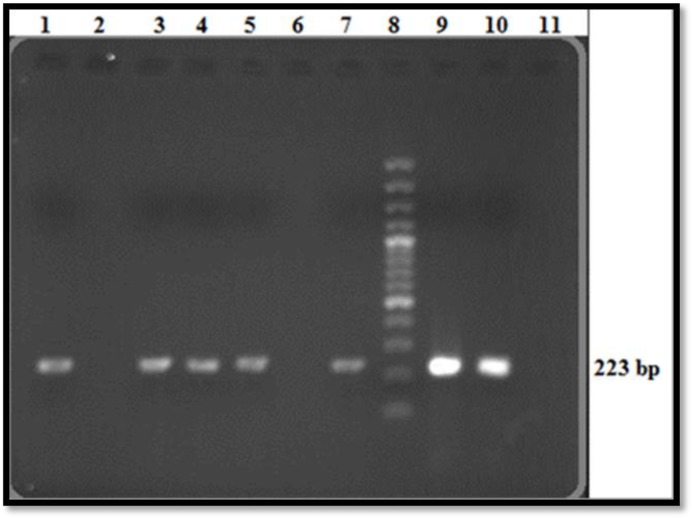
Analysis of PCR products on 2% agarose gel; Lane 1, 3, 4, 5, 7, Positive samples that clearly indicate a 223 bp PCR product; Lane 2, 6, Negative serum samples; Lane 8, 100 bp DNA ladder; Lane 9, 10, Positive controls; Lane 11, Negative control

Upon PCR analysis of the 100 serum samples, it was found that 45 serum samples (90%) that had positive tube agglutination results were also positive in PCR test (Figure 1). As expected, the 50 serum samples with negative tube agglutination and ELISA results were also negative in PCR. Interestingly, five (10%) samples that shown positive results in the tube agglutination test and four (8.16%) that were positive by ELISA, were negative when analyzed by PCR. A further study of PCR-positive results showed that all samples exhibited high titers of anti-*Brucella* antibodies (Table 1). One of the samples that was positive in the agglutination tube test was negative by both PCR and ELISA.

**Table 1:** Results of ELISA, agglutination, and PCR tests on serum samples

PCR Positive (no)	ELISA Positive (no)	Agglutination Positive (no)		Antibody titre in agglutination test
3	5		5	1/160
13	15		16	1/320
17	19		19	1/640
12	10		10	1/1280
45	49		50	**Total positive results**

## Discussion

Brucellosis is a zoonotic disease that afflicts both humans and animals. In certain cases, this infection can lead to a severe and prolonged illness in humans. As a result of this, rapid and reliable identification of the bacteria is necessary in order to initiate appropriate antibiotic treatment at the earliest. In order to, overcome limitations of the culture method for identification of the causative bacterium, various alternative methods have been used and introduced (8, 16-20). Molecular methods such as PCR have been reported as a tool to enable rapid and sensitive detection of this bacterium (11, 14, 15, 21). 

A study conducted by Maherand *et al.* has proposed that PCR should be regarded as the gold standard diagnostic method for detection of *Brucella* as this method had a higher sensitivity and specificity for detection as compared to other serological or culture-based methods (22,23). Mitka *et al.* concluded that the PCR amplification is efficient enough for the diagnosis of both acute as well as recurrent disease (23). In accordance with these findings, results of our study also clearly demonstrate that the PCR-based method had the ability to detect *Brucella* DNA from all patient samples that had IgM antibodies in serum. 

In the present study, it was found that 45 of the 50 serum samples with positive agglutination results had positive results from PCR too. All the 45 PCR-positive cases were also positive in ELISA, whereas four ELISA-positive samples were negative by PCR. These findings can be explained according to Navarro *et al.*, who established that if the results of clinical and serological methods prove brucellosis, negative results of blood culture and PCR-based methods should be considered as a false negative result (24). In another study, Gemechu *et al. *demonstrated that PCR has a lower sensitivity as compared to ELISA (13). Sensitivity and accuracy of PCR-based methods are dependent on the DNA extraction method and the quality of extracted genomic DNA (14). This method is also subject to inhibition by substances such as phenol, EDTA, DNase, RNase, etc. Several studies have reported the presence of bacterial DNA in both blood and serum samples. Zervaand *et al.* performed PCR on serum samples and found that in comparison to whole blood, serum also contains a sufficient quantity of DNA (14). In this study, *Brucella* DNA was extracted from serum samples, in four samples when analyzed by PCR no product was detected. This is in accordance with a study conducted by Ghorbani in 2013, wherein the authors observed that PCR was unable to detect* Brucella* DNA in three serum samples that have positive results in PCR analyses on whole blood samples (25). Previous studies explain the four negative results obtained by PCR method in our study. In an another study, Hajia *et al. *proposed that the cause of this problem due to the use of PCR in contrast to Real-time PCR (26). Another subject that should be considered while comparing detection methods, the sensitivity of PCR-based methods can be reduced in chronic brucellosis. This variation of sensitivity can account for four cases that had demonstrable IgG titers in the ELISA test but were negative in PCR. Another problem widely associated with the use of PCR was the possibility of contamination during processing of samples, which has the potential to lead to false positives, however, was not observed in our study. One case of serum sample that was positive by tube agglutination test was found to be negative in both ELISA as well as PCR methods; this can be attributed to the possibility of Gram-negative bacteria cross-reactivity in the serological agglutination test. In a study conducted in 2003 by Morata, was reported that the combined use of PCR and ELISA diagnostic tests can improve and overcome limitations in the diagnosis of brucellosis (21). The results obtained in this study confirm these finding. 

## Conclusion

Based on the results of this study, we propose that the simultaneous use of serology and molecular techniques has the potential to overcome limitations associated with the detection of Brucellosis.
